# Effect of Blanching Pomegranate Seeds on Physicochemical Attributes, Bioactive Compounds and Antioxidant Activity of Extracted Oil

**DOI:** 10.3390/molecules25112554

**Published:** 2020-05-31

**Authors:** Tafadzwa Kaseke, Umezuruike Linus Opara, Olaniyi Amos Fawole

**Affiliations:** 1 Postharvest Technology Research Laboratory, South African Research Chair in Postharvest Technology, Department of Food Science, Faculty of AgriSciences, Stellenbosch University, Private Bag X1, Stellenbosch 7602, South Africa; tafakaseqe@gmail.com; 2 Postharvest Technology Research Laboratory, South African Research Chair in Postharvest Technology, Department of Horticultural Sciences, Faculty of AgriSciences, Stellenbosch University, Private Bag X1, Stellenbosch 7602, South Africa; 3 Department of Botany and Plant Biotechnology, Faculty of Science, University of Johannesburg, P.O. Box 524, Johannesburg 2006, South Africa

**Keywords:** oil processing, fruit pomace, phytosterol, total phenolic compounds, carotenoids

## Abstract

This study investigated the effect of blanching pomegranate seeds (PS) on oil yield, refractive index (RI), yellowness index (YI), conjugated dienes (K232), conjugated trienes (K270), total carotenoid content (TCC), total phenolic compounds (TPC) and DPPH radical scavenging of the extracted oil. Furthermore, phytosterol and fatty acid compositions of the oil extracted under optimum blanching conditions were compared with those from the oil extracted from unblanched PS. Three different blanching temperature levels (80, 90, and 100 °C) were studied at a constant blanching time of 3 min. The blanching time was then increased to 5 min at the established optimum blanching temperature (90 °C). Blanching PS increased oil yield, K232, K270, stigmasterol, punicic acid, TPC and DPPH radical scavenging, whereas YI, β-sitosterol, palmitic acid and linoleic acid were decreased. The RI, TCC, brassicasterol, stearic acid, oleic acid and arachidic acid of the extracted oil were not significantly (*p* > 0.05) affected by blanching. Blanching PS at 90 °C for 3 to 5 min was associated with oil yield, TPC and DPPH. Blanching PS at 90 °C for 3 to 5 min will not only increase oil yield but could also improve functional properties such as antioxidant activity, which are desirable in the cosmetic, pharmaceutical, nutraceutical and food industries.

## 1. Introduction

Fruit processing generates huge amounts of waste in the form of compacted peels and seeds commonly referred to as pomace. This byproduct presents a huge disposal problem to the agro-processing industry as it is regarded as waste. However, fruit pomace is a good source of carbohydrates, proteins and lipids that are valuable for the production of human food supplements [1,2]. In addition, fruit pomace is a natural source of bioactive compounds such as dietary fiber, phenols, carotenoids, tocopherols, phytosterols and vitamins [3]. Commonly reported fruit pomace rich in bioactive compounds include grape, apple, strawberry, papaya and pomegranate [4,5,6,7].

The global production and consumption of pomegranate fruit continue to increase due to its health-promoting effects [8]. Consequently, this has generated more waste from the fruit between the preharvest and processing stages as sunburned, bruised, cracked and scalded fruit is rejected due to failure in meeting export market standards. Low quality grade fruit such as bruised fruit may be processed, further increasing losses in the form of peels and seeds. For instance, the pomegranate juice processing industry generates pomegranate pomace consisting of approximately 78% peels and 22% seeds [9,10]. Pomegranate seeds (PS) have attracted the interest of many researchers because they contain oil rich in punicic acid (65%–80%), a polyunsaturated fatty acid with multiple functional properties that are related to the prevention of coronary heart diseases, hypertension, diabetes, carcinogenesis, skin ageing and food decay [11,12,13,14]. With the increased consumer demand for natural food products for cases of chronic and degenerative diseases, valorisation of PS into oil is valuable to the food, cosmetic, nutraceutical and pharmaceutical industries.

Seed oil processing involves a series of steps that include drying, oil extraction and refining. Depending on the seed drying method and oil extraction technique, substantial amounts of bioactive compounds may be lost due to thermal degradation or extraction inefficiency as the bioactive compounds may remain trapped in the defatted seeds residues [15,16,17]. Cold pressing produces low seed oil yield, although the oil contains more natural and beneficial compounds such as tocopherols, sterols, carotenoids and phospholipids [18,19]. Supercritical carbon dioxide extraction, a greener seed oil extraction technique, is an expensive and energy intensive technology due to the high pressure needed to reach the super critical point, and therefore its application at a commercial level is limited [20]. Food and environmental safety concerns have been raised over the use of solvents such as hexane [21]. Furthermore, as hexane is non-polar, it is not a good solvent to extract polar bioactive compounds and therefore the extracted oil has poor functional properties compared to polar solvents such as ethanol [22].

The use of less toxic solvents such as ethanol in seed oil extraction has been studied. As a polar solvent, ethanol extracts polar antioxidant compounds that improve the functionality of the seed oil [23,24]. Furthermore, ethanol is cheaper, readily available, bio-renewable [25] and has been approved for food extraction by the European Directive 2009/32/EC [26]. The drawback with the use of ethanol is that it produces low oil yield [22,26]. To improve the oil extraction efficiency with ethanol, the seeds’ cellular structures may be altered prior to oil extraction to increase their permeability to lipids and bioactive compounds.

Researchers have studied several seed pretreatment techniques for improving oil yield and bioactive compound recovery such as enzyme digestion, microwave irradiation, ultrasound and pulse electric field exposure [27,28,29,30,31]. The highlighted seed pretreatment techniques for oil extraction are relatively expensive and their application at a commercial level is limited [32,33,34,35]. Blanching the oil seeds could be a promising technique to improve the yield and quality of the oil. It is an inexpensive technique that disintegrates the seeds’ cell walls and increases the extractability of the intracellular material [36]. This in turn reduces the oil extraction time and solvent and energy consumption. In addition, blanching cleans the seeds, inactivates the lipid oxidation enzymes, increases mass transport in the plant tissue and considerably reduces seed drying time [37,38].

To the best of our knowledge, the effect of seed blanching on oil yield and quality has not been studied. The objectives of this study were to investigate the effect of blanching PS on the quality of the extracted oil and establish the optimum blanching conditions for higher oil yield, better physicochemical attributes, bioactive compounds and antioxidant activity.

## 2. Results and Discussion

### 2.1. Oil Yield and Seeds Microstructure Changes

**Figure 1:**
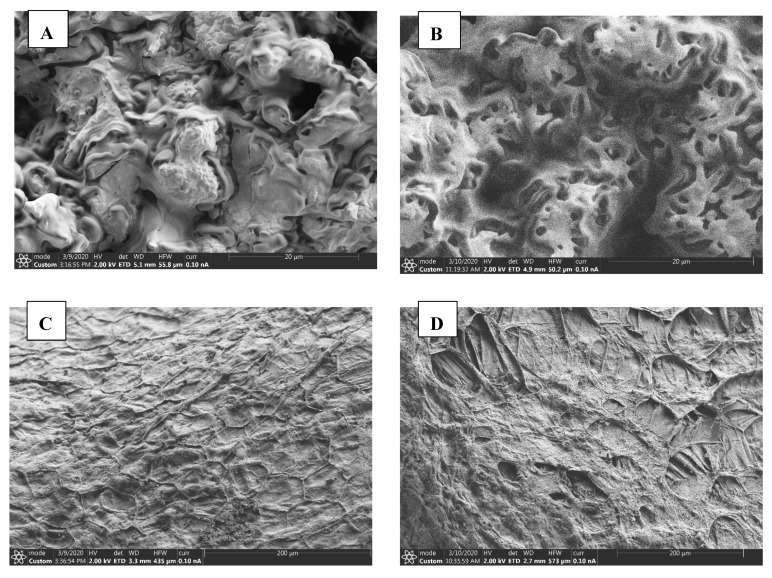
SEM graphs showing the effect of blanching (90 °C/3 min) on the pomegranate seed microstructures (**A**) unblanched pomegranate seeds, (**B**) blanched pomegranate seeds, (**C**) parenchymal cells from unblanched pomegranate seeds and (**D**) parenchymal cells from blanched pomegranate seeds.

The results in Table 1 and Table 2 demonstrate that increasing the blanching temperature and time significantly improved the pomegranate seed oil (PSO) yield (*p* < 0.05). Blanching PS at 90 °C for 3 min significantly increased the PSO yield by 35%, which was the highest improvement in the oil yield when compared with the oil yield from seeds blanched at 80 and 100 °C. Increasing the blanching time to 5 min at 90 °C significantly improved the oil yield by 18% when compared with unblanched PS, suggesting that blanching is a valuable pretreatment technique to enhance PSO yield. Furthermore, increasing the blanching temperature to 100 °C at a constant time of 3 min did not significantly increase the oil yield. To have a better insight on the effect of blanching on PS and the oil extraction mechanism, microscopic studies were done on both unblanched and blanched pomegranate seeds using a scanning electron microscope (SEM). The SEM images in Figure 1 confirm that blanching disintegrated the PS cell walls, loosening the hemi-cellulose, cellulose and pectin networks of PS [36,39]. Compared with unblanched PS, the SEM graph from blanched PS exhibited clear pores on the cells walls (Figure 1A,B). Figure 1C shows the parenchymal cells from unblanched PS with intact cell walls. In the absence of the blanching pretreatment, which provides cell disruption, the penetration of solvent into the cell matrix of the unblanched sample was limited as evidenced by the low oil yield from unblanched PS [30]. Figure 1D shows deformed parenchymal cells due to blanching pretreatment. The structural changes to the cell walls could have increased the permeability of the PS cell walls to the extraction solvent and enhanced the mass transfer of the lipids into the extraction solvent [40].

### 2.2. Refractive and Yellowness Index

Refractive index (RI) of seed oil is related to the degree of unsaturation and conjugation of the fatty acids and therefore can be used as an indirect way of measuring oil quality [41]. It was found that blanching PS did not significantly affect the RI of the extracted oil, despite significant effects of blanching PS on conjugated dienes, trienes and fatty acids (Table 1 and Table 2). Therefore, the interpretation of the RI results in this study should be done with caution. The RI of the extracted oil (1.5215–1.5218) was comparable to the one reported in literature. Costa et al. [42] reported RI ranging from 1.5091 to 1.5177 from cold pressed pomegranate seed oil.

The degree of seed oil yellowness commonly referred to as yellowness index (YI) is a valuable quality property of seed oil because color influences consumers’ choices and purchase decisions [43]. Blanching PS significantly decreased the oil YI (Table 1 and Table 2). Blanching PS at 80 and 90 °C for 3 min significantly reduced the YI by 22 and 17%, respectively. Slight but significant increase in YI (9%) was observed when the blanching temperature was raised to 100 °C. Increasing the blanching time to 5 min at a constant temperature of 90 °C significantly decreased the YI by 15% (Table 2). The decrease in the YI can be attributed to the oxidation and isomerisation of carotenoids compounds during blanching. In the presence of heat, *trans*-carotenoids (usual configuration) can be converted to *cis*-isomers, which may lead to the decrease in the oil yellowness [44]. Furthermore, blanching PS could have enhanced the extraction of Maillard reaction products that imparts a brownish color and reduces the oil yellowness [24,26].

### 2.3. Conjugated Dienes and Trienes

**Figure 2:**
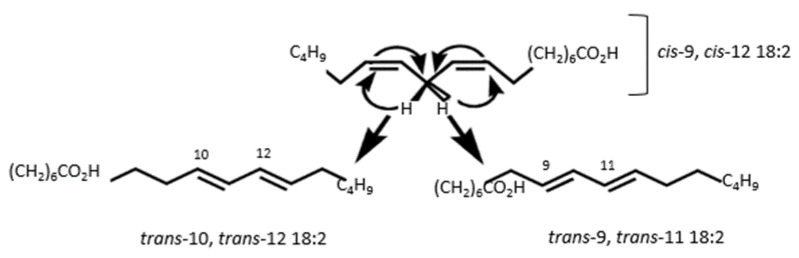
Formation of conjugated dienes and isomers of linoleic acid through sigmatropic rearrangement.

The seed oil conjugated dienes and trienes (K232 and K270) are a measure of the seed oil primary (conjugated dienes) and secondary products (conjugated trienes) of triglycerides oxidation [45]. Our findings demonstrate that blanching PS may increase the K232 and K270 values of the extracted oil (Table 1 and Table 2). For instance, blanching PS at 90 °C for 3 min significantly increased the K232 and K270 values 3- and 4-fold, respectively. As reported by Destaillats and Angers [46], the possible mechanism for the formation of conjugated bonds in the present study could be intramolecular sigmatropic rearrangement of the hydrogen atoms in polyunsaturated fatty acids such as linoleic acid to produce (*trans*-10, *trans*-12) and (*trans*-9, *trans*-11) conjugated linoleic acid isomers (Figure 2). Varying the blanching temperature did not significantly affect the K232 and K270 values, whilst these values significantly changed with variation in blanching time. Even though blanching PS significantly increased fatty acid conjugation, the K232 values (0.14–0.41) and K270 values (0.13–0.48) remained lower than those reported in previous studies, suggesting that blanching may not cause significant deterioration to the oil quality. Amri et al. [47] reported higher values of K232 values (4.15) from PSO hexane extracts of Tounsi cultivar. As shown in Figure 1, blanching may increase the porosity of the PS cell walls, promoting the leaching of antioxidative bioactive compounds and therefore exposing the fatty acids to oxidation [48]. In addition, Moghimi et al. [49] reported that the activity of lipolytic enzymes on fat and oil increases in damaged cells.

### 2.4. Total Carotenoids, Total Phenols and Antioxidant Activity

Carotenoids have been reported to prevent or reduce the risks of being affected by cardiovascular disease, age-related cataracts, macular degeneration and cancers [50,51]. As can be seen in Table 1 and Table 2, blanching PS neither significantly enhanced nor degraded the total carotenoid content (TCC) of the extracted oil. Carotenoids are insoluble in water and therefore could have resisted leaching during blanching [44]. In plant cells, carotenoids exist complexed together with proteins [31]. Although the SEM results in Figure 1 indicate that blanching deformed the PS cell walls, it could be hypothesized that the applied blanching temperature and time did not cause significant dissociation of these bioactive compounds from the protein complexes.

PSO is a good source of phenolic compounds and the total phenolic compounds (TPC) (2.01–5.58 mg GAE/g PSO) in the present study were higher than that of other fruit seed oils such as grape seed oil [48,52]. The results in Table 1 and Table 2 demonstrate that pretreatment of PS with blanching is a promising technique to enrich the phenolic content of the extracted oil. Increasing the blanching temperature from 80 to 90 °C at 3 min blanching time significantly improved the TPC from 3.03 to 4.04 mg GAE/g PSO. Further, increasing the blanching time to 5 min at a constant blanching temperature of 90 °C significantly increased the TPC by 1.5 mg GAE/g PSO (Table 2). In the seed cells, the phenolic compounds are bonded to other compounds to form complex structures [36]. Blanching PS could have reduced the complexation of phenolic compounds with the polysaccharides, proteins and pectin, thereby increasing their mass transfer into the oil phase. In addition, blanching PS could have initiated polymerization and increased the phenolic compounds [36]. Apart from enhancing the cell wall permeability, blanching may cause significant degradation or leaching of the phenolic compounds, a phenomenon that may explain the significant decrease in TPC (3.41–2.01 mg GAE/g PSO) when the PS was blanched at 100 °C for 3 min.

**Figure 3:**
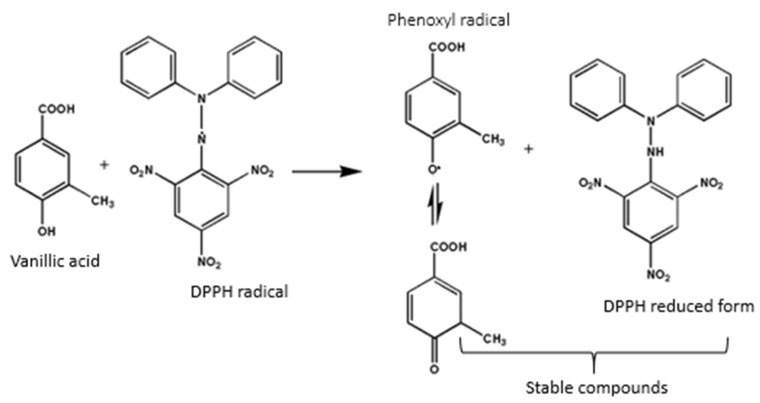
Mechanism of phenolic compound interactions with DPPH (2,2-Diphenyl-1-picrylhydrazyl) using vanillic acid as an example.

The impact of blanching PS on the antioxidant activity of PSO was evaluated using the DPPH assay. Blanching PS at 80 and 90 °C for 3 min significantly improved the DPPH radical scavenging of the extracted oil by 6% and 10%, respectively (Table 1 and Table 2). This could be directly related to the level of phenolic compounds recovered at these blanching conditions. The phenolic compounds scavenge the DPPH radical through hydrogen atom or electron donation (Figure 3). According to Koroleva et al. [53] the DPPH radical attack aims at the phenolic compound carbon atom with the highest electron density and subsequently abstracts the hydrogen atom from the hydroxyl group (OH) to a form stable diamagnetic molecule (2,2-Diphenyl-1-picrylhydrazine) and phenoxyl radical. The peroxyl radical is stabilized by the delocalized unpaired electrons created due to hydrogen abstraction. Increasing the blanching time to 5 min at 90 °C did not have a significant effect on the oil DPPH radical scavenging potential. When the blanching temperature was increased to 100 °C, the DPPH radical scavenging of the extracted oil significantly decreased by 6%. The results suggest that blanching PS at 90 °C for 3 min could be the optimum conditions for improved PSO antioxidant activity.

### 2.5. Phytosterols Composition

Phytosterols have similar biological functions as mammalian cholesterols, however they inhibit the absorption of mammalian cholesterol and therefore their intake from food of plant sources is important [54]. Three types of phytosterols including stigmasterol, β-sitosterol and brassicasterol were identified and quantified from oil of both unblanched and blanched PS. The results of phytosterol analysis in the PSO are presented in Table 3. Blanching PS (90 °C/3 min) significantly increased the stigmasterol by 1.9-fold but also significantly reduced β-sitosterol, the major phytosterol in PSO by 1.7-fold. Brassicasterol, the minor fatty acid identified in the PSO, was not significantly affected by blanching. The results suggest that the response to blanching differed among the phytosterols and the form in which they exist in the seed cells could explain the observation. In the plant cells, the phytosterols exist as free sterols or as conjugates of fatty acid esters and glycosides [53]. Since phytosterols are non-polar and insoluble in water, the possible loss of the β-sitosterol due to blanching could be thermal degradation. The decrease in β-sitosterol due to the seeds’ thermal pretreatment was also reported in previous studies. Zhou et al. [55] reported a 7% decrease in β-sitosterol of walnut oil microwaved at 600 W for 4 min.

### 2.6. Fatty Acids Composition

The results of fatty acid composition of PSO from unblanched and blanched seeds (90 °C/3 min) are shown in Table 4. The primary fatty acids identified in the PSO were punicic acid, linoleic acid, oleic acid, palmitic acid, stearic acid and arachidic acid. It was established that the effect of blanching PS on the fatty acid composition varied among the individual fatty acids. Blanching PS significantly decreased linoleic acid (18%), palmitic acid (16%) and saturated fatty acid (SFA) (16%) in the extracted oil. Nevertheless, the PSO from blanched seeds was significantly higher in punicic acid (7%), polyunsaturated fatty acid (PUFA) (3%) and UFA/SFA ratio (22%) than the oil from unblanched seeds. Since punicic acid (81.49%–83.83%) is the predominant component of the fatty acids, it can be suggested that the deformation of the PS cell walls increased its mass transfer into the extraction solvent (Figure 1). The significant increase in punicic acid in oil from blanched PS is essential because punicic acid is responsible for most of the biological activities of PSO, suggesting that the oil from blanched PS might still have improved functional properties regardless of the decrease in other fatty acids [13]. It is clear that blanching PS did not significantly affect the stearic acid, oleic acid, arachidic acid and monosaturated fatty acids (MUFA) of the extracted oil. Previous studies have also reported similar findings. For instance, Wroniak et al. [28], Moradi and Rahimi [29], Khittiphoom and Sutasinee [56] and Durdevic et al. [57] established that pretreatment did not significantly affect fatty acids in oils of rape, sunflower, mango and pomegranate seeds, respectively.

### 2.7. Principal Component Analysis

**Figure 4:**
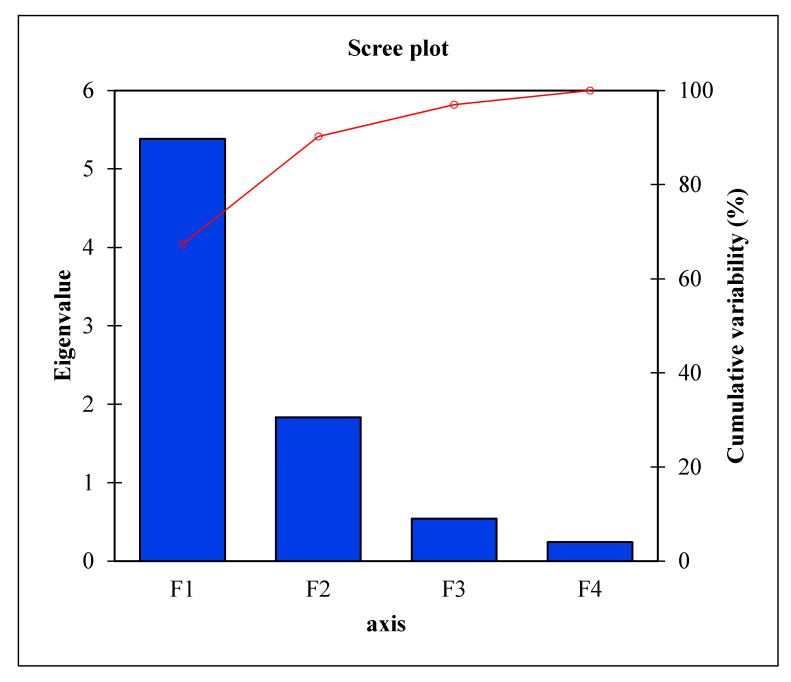
Scree plot of variance explained by each factor of the principal component.

**Figure 5:**
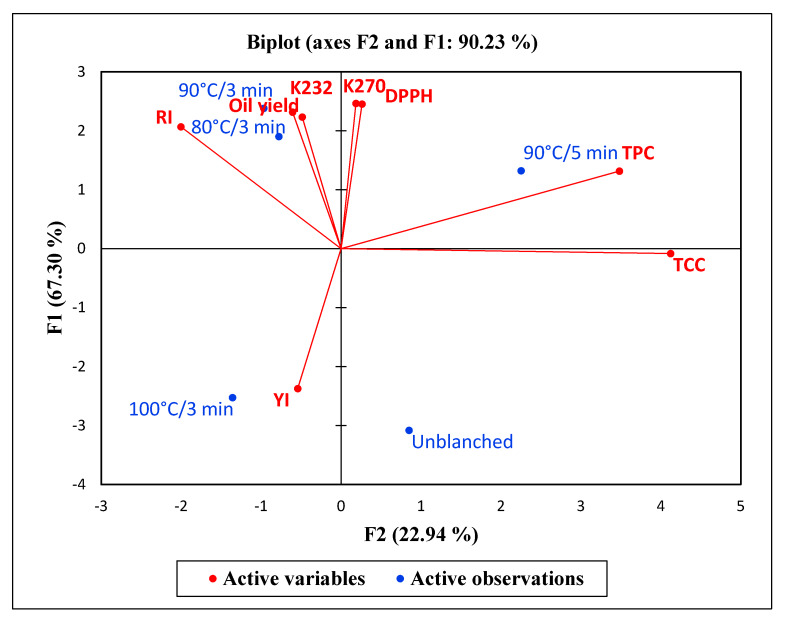
Principal component analysis data of PSO quality attributes from the different blanching conditions. YI = Yellowness index. RI = Refractive index. K232 = Conjugated dienes. K270 = Conjugated dienes TCC = Total carotenoids content. TPC = Total phenolic content. DPPH = 2.2-Diphenyl-1-picryl hydrazyl.

To have a better understanding of the influence of blanching conditions on the PSO quality, the multivariate data was subjected to principal component analysis (PCA). The eigenvalue measures the factor contribution to total variability. Eigenvalues greater than one are significant and therefore, the factors with the highest eigenvalues were considered the most important [58] (Figure 4). The first two principal components effectively separated the variables and observations and accounted for 90.23% of the total variation (Figure 5). The first factor (F1) was responsible for 67.30% of the total variation, whilst the second factor (F2) explained 22.94% of the total variation, demonstrating that the maximum possible variation in the oil quality due to blanching was explained by the F1. Positive scores on F1 corresponded with oil from PS blanched at 90 °C for 3 min. These variables were associated with oil yield and DPPH radical scavenging ability in addition to K232, K270 and RI. The higher oil yield obtained at this particular blanching condition could mean that higher amounts of K232 and K270 were proportionally generated, subsequently increasing the RI of the oil [41]. Negative scores on F1 corresponded with oil from unblanched PS as well as oil from PS blanched at 100 °C for 3 min and were associated with YI. This illustrates that improvements in oil yield and antioxidant activity were associated with decreases in the oil YI a phenomenon that can be explained by increased extractability of phenolic compounds and oxidation of color pigments such as carotenoids. Positive scores on F2 corresponded to oil from PS blanched at 90 °C for 5 min, that were correlated with TPC and TCC.

## 3. Materials and Methods

### 3.1. Materials

Commercially mature pomegranate fruits (cv. Wonderful) were procured from Sonia pack-house (33°34′851″ S, 19°00′360″ E) in Western Cape Province, South Africa. The fruits were stored in the Postharvest Technology Research Laboratory at Stellenbosch University at 7.5 ± 0.5 °C and 92% ± 3% RH prior to processing to preserve quality [59]. The chemical reagents used in the current study including Folin–Ciocalteau reagent, gallic acid, β-Carotene, heptadecanoic acid, 2,4,6-Tri(2-pyridyl)-s-triazine (TPTZ), 6-hydroxy-2,5,7,8-tetramethylchromane-2-carboxylic acid (Trolox), 5α-Cholestan-3β-ol and 2,2-Diphenyl-1-picrylhydrazyl (DPPH), were supplied by Sigma, South Africa. All the other solvents and reagents were of analytical grade.

### 3.2. Sample Preparation and Blanching Pretreatments

Pomegranate seeds (PS) were collected from the fruit pomace after juice extraction using a hand juice-pressing machine. Clean PS were hot water blanched in a water bath (Scientific, Maraisburg, South Africa) at 80, 90 and 100 °C for 3 min. The blanching time of 3 min was based on a preliminary experiment, which confirmed that blanching below 3 min, regardless of temperature, did not have a significant effect on oil yield. In order to establish the effect of an increase in blanching time on oil yield and quality attributes, a follow-up experiment was carried at the optimum temperature (90 °C) for 5 min. The blanched seeds were quickly cooled with cold water to stop the blanching process after which they were oven dried at 55 ± 2 °C for 24 h to 2% (*w*/*w*) moisture content [60]. Moisture content of the dried seeds was determined in a moisture analyzer at 100 °C (KERN, DBS60-3, Balingen, Germany). The dried seeds were stored at 4 ± 2 °C until oil extraction [61].

### 3.3. Oil Extraction

Ultrasound-assisted solvent extraction was used to extract the oil from ground PS (<1 mm particle size) following the method described by Samaram et al. [62] with minor modifications. An ultrasonic bath (Scientific, South Africa) (maximum power: 700 W, frequency: 40 kHz and internal dimensions: 500 mm × 300 mm × 150 mm) was used to extract the oil. The ultrasound extraction was carried out under the following experimental conditions: temperature (40 ± 5 °C), time (40 min), solid to solvent ratio (1:5 *w*/*v*) and maximum power (40 MHz and 700 W). The samples (20 g) were mixed with ethanol, sonicated and filtered under vacuum before they were subjected to vacuum evaporation to recover the solvent (G3 Heidolph, Germany). Unblanched PS powder was used as the control sample. Oil extraction was done twice after which the samples were packed in brown bottles and stored at 4 ± 2 °C [21]. The extraction was performed in triplicates (*n* = 3). The pomegranate seed oil (PSO) yield was calculated gravimetrically as:

(1) $$\mathrm{PSO}\;\mathrm{yield}\;\left(\%\right)=\frac{M_{1}}{M_{2}}\times 100$$

where M_1_ is the mass of the PSO and M_2_ is the dry mass of the pomegranate seeds.

### 3.4. Pomegranate Seeds Microstructure Analysis

A field emission scanning electron microscope (FESEM) (Thermo Fisher Apreo, Waltham, MA, USA) was used to investigate the changes in the PS microstructures due to blanching treatment. The sample was mounted on aluminum stubs with double-sided carbon tape and then sputter-coated with a thin layer of gold (10 nm thick) using a gold sputter coater (EM ACE200, Leica, Wetzlar, Germany) to make the sample conductive. The images were collected using a voltage of 2 kV and recorded digitally.

### 3.5. Physicochemical Attributes Analysis

#### 3.5.1. Yellowness and Refractive Index

Refractive index (RI) was measured at ambient conditions (25 °C) using a calibrated Abbe 5 refractometer (Bellingham + Stanley, United Kingdom). Colour (L*: lightness and b*: yellowness) of the extracted PSO was determined using a calibrated Chroma meter CR-410 (Konica Minolta, INC, Tokyo, Japan). Yellowness index (YI) was calculated as:

(2) $$\mathrm{YI}=\frac{142.86b^{*}}{L^{*}}$$

#### 3.5.2. Conjugated Dienes and Trienes

PSO conjugated dienes (K232) and trienes (K270) were determined according to the standard [63]. The oil solution (1%) (*w*/*v*) was prepared by dissolving it in cyclohexane. The absorbance values were measured at 232 and 270 nm with a UV Spectrophotometer (Thermo Scientific technologies, Madison, WI, USA).

### 3.6. Bioactive Compounds and Antioxidant Activity Determination

#### 3.6.1. Total Carotenoids

PSO (0.2 g) was dissolved in hexane (2 mL) and 0.5 mL of 0.5% (*w*/*v*) sodium chloride (NaCl) was added. The mixture was vortexed for approximately 30 s and centrifuged (Centrifuge 5810R, Eppendorf, Hamburg, Germany) for 10 min at 1500 rpm. Absorbance of the supernatant was measured at 460 nm with a UV spectrophotometer (Helios Omega, Thermo Scientific, Waltham, MA, USA). The absorbance of the β-carotene standard was linear between 0.5–100 µg/mL. Results were expressed as mgβ-carotene/100 g of PSO.

#### 3.6.2. Total Phenolic Compounds

Total phenolic content of PSO was determined according to a method described by Abbasi et al. [11]. The reaction mixture contained 100 µL of PSO extracts, 500 µL of the Folin–Ciocalteau reagent, 1.5 mL of 20% (*w*/*v*) sodium carbonate and 6 mL of distilled water. The solutions were incubated in the dark for 30 min and their absorbance measured at 760 nm with a UV Spectrophotometer (Helios Omega, Thermo Scientific, Waltham, MA, USA). Gallic acid standard curve (0.0–20 mg/mL) was used and the total phenolic compounds were expressed as milligram gallic acid equivalent per mL PSO (mg GAE/g PSO).

#### 3.6.3. Phytosterol Composition

PSO (0.1 g) was weighed into 15 mL glass vials and 2.5 mL of saponification reagent (94 mL of absolute ethanol, 6 mL of 33% (*w*/*v*) potassium hydroxide, 500 µL of 20% (*w*/*v*) ascorbic acid) was added. To the samples, 100 µL of 5 α-cholestenol (1000 ppm) in chloroform was added as an internal standard. The mixture was vortexed and then saponified in an oven at 60 °C for 1 h. The saponified samples were cooled in ice for 10 min after which 5 mL of distilled water and 2 mL of chloroform were added and the mixture vortexed. The samples were centrifuged at 3000 rpm for 4 min. The chloroform extracts (500 µL) were transferred into 2 mL glass vials and concentrated with a gentle stream of nitrogen to ± 200 µL. To 100 µL of the chloroform extracts, pyridine (100 µL) and *N*,*O*-Bis (trimethylsilyl) trifluoroacetamide (30 µL) were added and the mixture was vortexed and then derivatized in an oven at 100 °C for 1 h. The derivatized samples were analyzed with GC-MS. The results were expressed as mg/100 g of PSO.

#### 3.6.4. Antioxidant Activity

The antioxidant activity of PSO was determined using 2, 2-Diphenyl-1-picryl hydrazyl (DPPH) following the procedure described by Siano et al. [64]. In brief, 0.1 mL aliquot of oil extract was added to 2.5 mL of 0.0004% (*w*/*v*) freshly prepared DPPH in 80% (*v*/*v*) methanol. The mixture was vortexed and immediately incubated in the dark for 60 min. The absorbance was measured with a UV spectrophotometer (Helios Omega, Thermo Scientific, Waltham, MA, USA) at 517 nm. The absorbance of DPPH in 80% methanol was measured as the negative control. Lower absorbance represented higher radical scavenging ability [65]. The absorbance of the Trolox standard curve was linear between 5–100 mM. Results were reported as mmol Trolox/g of PSO.

### 3.7. Analysis of Fatty Acid Composition

The fatty acid composition of PSO was determined by the gas chromatography-mass spectrometry (GC-MS) method as described by Mphahlele et al. [66]. Briefly, pomegranate seed oil (0.1 g) was weighed into 15 mL glass vials followed by the addition of 2.0 mL hexane, 50 µL heptadecanoic acid (1000 ppm, internal standard) and 1.0 mL of 20% (*v*/*v*) H_2_SO_4_ in methanol. The reaction mixture was vortexed and incubated at 80 °C for 1 h in an oven. The mixture was cooled and then 3 mL of saturated NaCl was added, vortexed and centrifuged. The supernatant containing hexane phase was transferred into vials for analysis with GC-MS (6890N, Agilent technologies network) coupled to an Agilent technologies inert XL EI/CI Mass Selective Detector (MSD) (5975B, Agilent technologies Inc., Palo Alto, CA, USA). Helium was employed as the carrier gas at a flow rate of 0.017 mL/s. One microliter of sample was injected in a split ratio of 10:1. The oven temperature was run as: 100 °C/min, 180 °C at 25 °C/min and held for 3 min, 200 °C at 4 °C/min and held for 5 min, 280 °C at 8 °C/min, and 310 °C at 10 °C/min and held for 5 min. The pomegranate seed oil fatty acid profiles were identified using the NIST library. Results were expressed as mean area % relative abundance.

### 3.8. Statistical Analysis

The results of all the studied variables were presented as mean ±S.E (standard error). Significant differences were calculated using the independent t-test and one way analysis of variance (ANOVA) (*p* < 0.05), using Statistica software (Statistical v13, TIBC, Palo Alto, CA 94304, USA) and the mean values were separated according to Duncan’s multiple range test. The relationship between the variables and observations was determined by performing principal component analysis (PCA) using Microsoft Excel (XLSTAT 2019.4.1.63305, Addinsoft, New York, NY, USA).

## 4. Conclusions

The valorization of PS into oil is a valuable way of managing the pomegranate waste generated during processing. The present study showed that blanching PS increased oil yield and K232, K270, stigmasterol, punicic acid, TPC and antioxidant activity. However, it decreased the YI, β-sitosterol, palmitic acid and linoleic acid of the oil. Regarding RI, TCC, brassicasterol, stearic acid, oleic acid and arachidic acid, blanching PS did not present any significant effect. PS blanched at 90 °C for 3 to 5 min significantly improved the oil yield, TPC and DPPH radical scavenging. With the significant improvement in TPC and antioxidant activity of the oil from blanched PS, it can be concluded that blanching PS as a pretreatment technique for oil extraction may improve the antioxidant properties of the oil, an attribute that is valuable to the oil for its application in the cosmetic, pharmaceutical, nutraceutical and food industries.

## Figures and Tables

**Table 1:** Physicochemical and bioactive compounds and antioxidant activity of oil from pomegranate seeds blanched for 3 min as a function of blanching temperature.

Blanching Temperature (°C)	Oil Yield (%)	YI	K_232_	K_270_	RI	TCC	TPC	DPPH
Unblanched	13.70 ± 0.10 ^c^	83.86 ± 0.13 ^a^	0.14 ± 0.01 ^c^	0.13 ± 0.02 ^c^	1.5215 ± 0.00 ^a^	30.12 ± 1.07 ^a^	3.41 ± 0.05 ^a^	4.06 ± 0.11 ^c^
80	15.80 ± 0.60 ^b^	65.47 ± 0.30 ^c^	0.41 ± 0.02b ^b^	0.48 ± 0.02 ^b^	1.5218 ± 0.00 ^a^	28.74 ± 1.07 ^a^	3.03 ± 0.17 ^c^	4.32 ± 0.05 ^ab^
90	18.55 ± 0.55 ^a^	67.04 ± 0.06 ^b^	0.32 ± 0.04 ^a^	0.41 ± 0.01 ^a^	1.5218 ± 0.00 ^a^	26.79 ± 1.12 ^a^	4.04 ± 0.08 ^d^	4.41 ± 0.07 ^b^
100	14.20 ± 0.20 ^bc^	91.52 ± 0.16 ^d^	0.16 ± 0.00 ^c^	0.15 ± 0.02 ^c^	1.5217 ± 0.00 ^a^	27.74 ± 0.10 ^a^	2.01 ± 0.07 ^b^	4.13 ± 0.03 ^ac^

Values are means ± SE of triplicate determinations. Means in the same column and followed by different letters are significantly different (*p* < 0.05) according to Duncan’s multiple range test. RI = Refractive index (25 °C), YI = Yellowness index, K232 = Conjugated dienes, K270 = Conjugated trienes, TCC = Total carotenoids content (mg β-carotene/100 g PSO), TPC = Total phenolic content (mg GAE/g PSO), DPPH = 2.2-Diphenyl-1-picryl hydrazyl (mmol Trolox/g PSO), GAE = Gallic acid equivalence, PSO = Pomegranate seed oil.

**Table 2:** Physicochemical and bioactive compounds and antioxidant activity of oil from pomegranate seeds blanched at 90 °C as a function of blanching time.

Blanching Time (min)	Oil Yield (%)	YI	K_232_	K_270_	RI	TCC	TPC	DPPH
Unblanched	13.70 ± 0.10 ^b^	83.86 ± 0.13 ^c^	0.14 ± 0.01 ^a^	0.13 ± 0.02 ^a^	1.5215 ± 0.00 ^a^	30.12 ± 1.07 ^ab^	3.41 ± 0.05 ^b^	4.06 ± 0.11 ^a^
3	18.55 ± 0.55 ^c^	67.04 ± 0.06 ^b^	0.32 ± 0.04 ^b^	0.41 ± 0.01 ^b^	1.5218 ± 0.00 ^a^	26.79 ± 1.12 ^b^	4.04 ± 0.08 ^a^	4.44 ± 0.06 ^b^
5	16.23 ± 0.13 ^a^	71.05 ± 0.19 ^a^	0.28 ± 0.04 ^b^	0.41 ± 0.01 ^b^	1.5217 ± 0.00 ^a^	32.94 ± 2.49 ^a^	5.58 ± 0.07 ^c^	4.36 ± 0.05 ^b^

Values are means ± SE of triplicate determinations. Means in the same column and followed by different letters are significantly different (*p* < 0.05) according to Duncan’s multiple range test. RI = Refractive index (25 °C), YI = Yellowness index, K232 = Conjugated dienes, K270 = Conjugated trienes, TCC = Total carotenoids content (mg β-carotene/100 g PSO), TPC = Total phenolic content (mg GAE/g PSO), DPPH = 2.2-Diphenyl-1-picryl hydrazyl (mmol Trolox/g PSO), GAE = Gallic acid equivalence, PSO = Pomegranate seed oil.

**Table 3:** Phytosterol composition (mg/100 g pomegranate seed oil, PSO) of oil from the unblanched and blanched pomegranate seeds at optimum conditions.

Phytosterol	Unblanched	90 °C/3 min
Stigmasterol	4.02 ± 0.57 ^a^	7.59 ± 1.00 ^b^
β-Sitosterol	949.09 ± 18.87 ^b^	571.73 ± 23.55 ^a^
Brassicasterol	1.99 ± 0.07 ^a^	2.22 ± 0.01 ^a^

Values are means ± SE of duplicate determinations. Means in the same row and followed by different letters are significantly different (*p* < 0.05) according to Duncan’s multiple range test.

**Table 4:** Fatty acid composition and their relative abundance (%) in oil from the unblanched and blanched pomegranate seeds at optimum conditions.

Fatty Acid	Unblanched	90 °C/3 min
Palmitic (C16:0)	6.70 ± 0.015 ^b^	5.61 ± 0.14 ^a^
Stearic (C18:0)	2.64 ± 0.09 ^a^	2.37 ± 0.04 ^a^
Oleic (C18:1)	7.18 ± 0.09 ^a^	6.72 ± 0.15 ^a^
Linoleic (C18:2)	12.59 ± 0.40 ^b^	10.28 ± 0.35 ^a^
Punicic (C18:3)	68.91 ± 0.77 ^a^	73.55 ± 0.36 ^b^
Arachidic (C20:0)	0.68 ± 0.08 ^a^	0.46 ± 0.02 ^a^
ƩSFA	10.02 ± 0.27 ^b^	8.44 ± 0.16 ^a^
ƩMUFA	7.18 ± 0.09 ^a^	6.72 ± 0.15 ^a^
ƩPUFA	81.49 ± 0.37 ^a^	83.83 ± 0.06 ^b^
UFA/SFA ratio	8.86 ± 0.26 ^a^	10.74 ± 0.22 ^b^

Values are means ± SE of triplicate determinations. Means in the same row and followed by different letters are significantly different (*p* < 0.05) according to Duncan’s multiple range test. SFA = Saturated fatty acid, MUFA = Monounsaturated fatty acid, PUFA = Polyunsaturated fatty acid, UFA = Unsaturated fatty acid.
